# Nerve transfers for brachial neuralgia

**DOI:** 10.1186/1753-6561-9-S3-A28

**Published:** 2015-05-19

**Authors:** Somsak Leechavengvongs

**Affiliations:** 1 Institute of Orthopaedics, Lerdsin General Hospital, Bangkok, 10500, Thailand

## 

Motor function, rather than sensory function, is the primary concern for most brachial plexus surgeons. After root avulsions from the spinal cord, numbness in the affected limb may be associated with extreme, almost unbearable, intractable pain. Deafferent pain is the result of avulsion of the root from the spinal cord. Pain from the brachial plexus avulsion probably arises from the disinhibition of neurons within the substantia gelatinosa that fire spontaneously. The convulsive pain of the brachial plexus injury may correlate with the burst pattern of firing in lamina V also observed in higher levels of the central nervous system including the thalamus. It has been postulated that the lack of sensory input into the spinal cord leads to spontaneous discharges of the neurons in the dorsal horn, therebycausing pain.

In our experience, one-third of patients with C5 and C6 root avulsion presented with pain on the dorsal radial aspect of the hand correlated with the superficial radial nerve distribution. Nerve transfer may inhibit abnormal electrical activity in the dorsal horn or higher levels of the central nervous system such as the thalamus by restoring the input to the damaged limb.

In 2011 we described the technique “End-to-Side Radial Sensory to Median Nerve Transfer to Restore Sensation and Relieve Pain in C5 and C6Nerve Root Avulsion” in 8 patients. Six patients had S2 and 2 patients had S3. All patients perceived at least one number lower of the Semmes-Weinstein filament in the dorsal radial aspect of the affected hand compared with the preoperative status. The best result was perception of the 3.61 filament in 2 patients. No downgrading of the donor nerve was observed after surgery. All patients had relief of pain 2 weeks after surgery, and the pain decreased dramatically at the last follow-up.

An interesting finding in our patients is that we observed no cross-misperception between the injured nerve and donor nerve territories. One hypothesis is that the induced collateral axons are specifically aimed to their new target reconnecting with the spinal cord neurons; another explanation is that there is an unknown mechanism of brain plasticity.

Recently, we performed end-to-side median nerve into contralateral C7 transfer to relieve pain at the median nerve distribution area of the hand in late total arm type brachial plexus injury in 7 patients. All patients perceived at least 2 number lower of the Visual Analog Scale (VAS) for pain at the median nerve distribution area of the affected hand and at least 20% decrease in the PSRS compared with the preoperative status. The mean VAS was decreased from 7.8 to 4.1 and the mean PSRS was decrease from 28.4 to 17.5 at last F/U. No downgrading of the donor nerve was observed after surgery. None of the patients experienced any major complication. This nerve transfer could be an alternative method to treat refractory deafferent pain in total arm type brachial plexus injury without major complications.

